# Antioxidant Potential of Selected Wild Edible Leafy Vegetables of Sikkim Himalayan Region: Effects of Cooking Methods and Gastrointestinal Digestion on Activity

**DOI:** 10.3389/fnut.2022.861347

**Published:** 2022-04-21

**Authors:** Swati Sharma, Srichandan Padhi, Megha Kumari, Srinivas Patnaik, Dinabandhu Sahoo

**Affiliations:** ^1^Institute of Bioresources and Sustainable Development, Regional Centre, Gangtok, India; ^2^School of Biotechnology, Kalinga Institute of Industrial Technology, Bhubaneswar, India; ^3^Department of Botany, University of Delhi, New Delhi, India

**Keywords:** green leafy vegetables, phenolic contents, antioxidant activity, *Urtica dioica*, Sikkim Himalaya

## Abstract

Green leafy vegetables or GLVs are one of the main attractions in the local vegetable market and are widely consumed as the main course and side dish in the Sikkim Himalayan region (SHR). This study evaluated the total phenolic (TPC) and flavonoid contents (TFC) and antioxidant potential in different extracts such as methanolic (MeOH), ethyl acetate (EtOAC), and hexane extracts of selected GLVs followed by changes in the antioxidant activity on cooking and stimulated gastrointestinal (GI) digestion. The MeOH extracts of *Urtica dioica* L. (Sisnu), *Nasturtium officinale* W. T. Aiton (Simrayo), *Diplazium esculentum* Retz. Sw. (Ningro), and *Chenopodium album* L. (Bethu) were estimated to have higher TPC [22.73–45.84 μg gallic acid equivalent (GAE)/mg of extract]. In contrast, the plant extracts prepared using EtOAC (except for *N. officinale*, where TFC was found to be higher in hexane extract) were found to contain higher TFC (3.42–14.86 μg quercetin equivalent (QE)/mg of extract). The MeOH extracts also exhibited higher 2, 2-diphenyl-1-picrylhydrazyl (DPPH) scavenging activity (9.55–18.67 μg ascorbic acid equivalent (AAE)/mg of extract), total antioxidant activity (TAA) (0.27–0.32 mg AAE/mg of extract), and reducing power potential (RPP) (1.6–9.9 μg AAE/mg of extract). Among the test MeOH extracts, *U. dioica* demonstrated relatively higher antioxidant activities and was selected for cooking experiments followed by simulated GI digestion. The findings revealed that the loss of antioxidant activity was minimal in steam-cooked leaves (3.5% in 40 min) as compared to the boiled ones (18% in 10 min). The simulated GI (simulated salivary, gastric, and intestinal) digestion performed on raw, steam cooked, and boiled *U. dioica* leaves showed substantial enhancement of antioxidant properties (by 64.63%) through steam cooking in comparison to the raw leaves. Overall the study concludes that higher antioxidant properties can be achieved on the consumption of steam-cooked *U. dioica* leaves.

## Introduction

Reactive oxygen species (ROS) which include free radicals such as hydrogen peroxide (H_2_O_2_), superoxide (${\text{O}}_{2}^{-}$), hydroxyl radical (OH–), singlet oxygen (^1^O_2_), and alkoxyl radical (RO) are formed continuously in the human body because of different cellular metabolic processes (electron transport chain, phagocytosis, fertilization, energy production in mitochondria, etc.) (1–4). These ROS are reported to cause oxidative damage to living cells on reacting with different molecules of the cell and are responsible for the development of several pathological conditions including DNA damage, lipid peroxidation, protein oxidation, and cellular degeneration. Moreover, uncontrolled generation of ROS has also been associated with various diseases and disorders such as diabetes, cancer, rheumatoid arthritis, osteoporosis, and aging (1, 5). The human body has developed an endogenous defense system consisting of a few antioxidant enzymes (catalase, glutathione reductase, superoxide dismutase, etc.) to protect itself from the harmful effects of ROS; nevertheless, it fails to be effective against the oxidant load in certain conditions. Therefore, the human body needs to be supplied with exogenous antioxidants.

Antioxidants are small molecules that can help to limit or prevent the harmful effects of pervasive free radicals. These molecules are highly essential for the survival of all living beings. Plant-based foods are an excellent source of natural antioxidants such as flavonoids and related phenolic compounds that offer a balance between the oxidants and antioxidants in the body and thereby play role in combating the oxidative stress inside the human body (6). The green leafy vegetables or GLVs which are being commonly used in every household as a part of healthy and promising meals contain a blend of natural antioxidants and phenolic compounds that render them exceptional for several nutritional and important therapeutic properties (7–9). Studies have shown that regular consumption of GLVs rich in antioxidants results in a positive impact by decreasing oxidative damage (10). Conversely, before their consumption, most of the GLVs need to be undergone a cooking process (boiling, steaming, frying, etc.) which is based on taste preference and edibility. Cooking can also induce changes in the physical characteristics, the chemical composition of the food, and influence the concentration, release, and bioavailability of bioactive substances (11, 12). In certain cases, specific culinary methods have resulted in the denaturation of active constituents and thereby reducing the nutritional and therapeutic values. Specific cooking processes have also been shown to enhance the functional properties of selected GLVs (1, 13, 14). The extent of benefits anticipated from foods can also be influenced by the physiological process of digestion where the digestive enzymes act while the food travels through the gastrointestinal (GI) tract (13, 15).

Sikkim, a small Himalayan state of northeastern India, is known for its multiethnic population and notable biodiversity including subtropical and alpine climates. The wild edible leafy vegetables have been valued greatly throughout this region and serve as sources of food for the inhabitants. The mode of consumption and ethnopharmacological significance of some of these vegetables have also been documented (16). Nonetheless, the data on the effects of different cooking processes on the bioactivity of such GLVs are still limited. In addition, the fates of bioactive components during the process of GI digestion have merely been studied. Therefore, this study was aimed at evaluating the antioxidant potential of organic extracts prepared from selected green leafy vegetables commonly used in the Sikkim Himalayan region (SHR), followed by investigating the effects of different cooking methods and *in-vitro* GI digestion on retention of their antioxidant properties. The outcome of the study may contribute to the promotion of consumption of GLVs in SHR and other regions of the country to use a selective cooking approach to assure better antioxidant benefits.

## Materials and Methods

### Materials

Methanol (MeOH), ethyl acetate (EtOAC), hexane, sodium carbonate (Na_2_Co_3_), Folin–Ciocalteu reagent, aluminum chloride (AlCl_3_), potassium acetate (CH_3_COOK), sulfuric acid (H_2_SO_4_), sodium phosphate (Na_3_PO_4_), ammonium molybdate [(NH_4_)_2_MoO_4_], 2,2-diphenyl-1-picrylhydrazyl (DPPH), potassium ferricyanide K_4_[Fe(CN)_6_], trichloracetic acid (TCA), potassium chloride (KCl), sodium bicarbonate (NaHCO_3_), potassium dihydrogen phosphate (KH_2_PO_4_), ammonium carbonate [(NH_4_)_2_CO_3_], sodium chloride (NaCl), hydrochloric acid (HCl), magnesium chloride (MgCl_2_.6H_2_O), α-amylase, pepsin, pancreatin, calcium chloride (CaCl_2_), and sodium hydroxide (NaOH) are from Sigma Aldrich.

### Collection of Plant Samples and Preparation of Extracts

Green leafy vegetables (GLVs) used in this study including *Urtica dioica, Nasturtium officinale, Diplazium esculentum*, and *Chenopodium album* were purchased from Lal Bazaar, a local market under Gangtok Municipal Corporation, East Sikkim. Collected leaves were washed in running tap water, oven-dried (40°C), and powdered using a mixer grinder. A definite quantity of leaf powders was extracted with 1:5 (w/v) of three different organic solvents of varying polarity (methanol, ethyl acetate and hexane) using cold maceration. Briefly, the leaf powders (100 g) were dissolved differently in 500 ml of the organic solvent and shaken vigorously for 15 min. Then, the solutions were kept in dark and shaking was performed at every 12 h interval. After 72 h, the solutions were filtered using Whatman No. 1 filter paper and the solvents were made evaporated using Rota evaporator (R 100, Buchi) to obtain the organic extracts which were stored at 4°C for further use.

### Estimation of Total Phenolic Content

Total phenolic content (TPC) of the organic extracts was estimated following the Folin–Ciocalteu method previously described by Rai et al. (17). Briefly, 50 μl (2.5 mg/ml) extract was mixed with 2 ml of 2% Na_2_CO_3_ followed by incubation at room temperature for 2 min. Thereafter, 100 μl of 50% Folin–Ciocalteu reagent was added to the reaction mixture and incubated at room temperature for 30 min. Absorbance was recorded at 720 nm using a UV-VIS spectrophotometer (Shimadzu, Kyoto, Japan). All the analyses were performed in triplicates and data were expressed in terms of μg gallic acid equivalent (GAE)/mg of the organic extract.

### Estimation of Total Flavonoid Contents

Total flavonoid contents (TFCs) of the organic extracts were determined following a standard protocol as described by Aryal et al. (18). To 1 ml (0.625 mg/ml) extract, 0.2 ml of 10% (w/v) AlCl_3_ (in MeOH) and 0.2 ml (1 M) potassium acetate were added. The volume of the reaction mixture made up to 7 ml by adding distilled water and incubated at room temperature for 30 min. The absorbance was measured at 415 nm using a UV-VIS spectrophotometer. The data were expressed in terms of μg quercetin equivalent (QE)/mg of extract.

### Determination of Total Antioxidant Activity

Total antioxidant activity (TAA) of the leaf extracts was determined following the method described by Rai et al. (19). Briefly, 200 μl (0.25 mg/ml) of extract was mixed with 3 ml reagent solution prepared using 0.6 M sulfuric acid, 28 mM sodium phosphate, and 4 mM ammonium molybdate (1:1:1). The components of the mixture were mixed well and incubated at 95°C in the water bath for 90 min. The reaction mixture was then allowed to cool to room temperature and the absorbance was recorded at 695 nm using a UV-VIS spectrophotometer. The data were expressed in mg ascorbic acid equivalent (AAE)/g of extract.

### DPPH Radical Scavenging Activity

The leaf extracts were determined for their DPPH radical scavenging activity using the method described by Rai et al. (20). Briefly, 200 μl (1.25 mg/ml) extracts were mixed with 2 ml 0.16 mM DPPH solution followed by incubation in a dark condition for 30 min. After incubation, the absorbance of content was taken at 517 nm using a UV-VIS spectrophotometer. The DPPH scavenging activity was calculated using the following equation:


$$\begin{array}{l} \text{Scavenging effect }\left(\text{\%}\right)=\left\{1-\left({\text{S}}_{\text{abs}}-{\text{B}}_{\text{abs}}/{\text{C}}_{\text{abs}}\right)\right\}\times 100 \end{array}$$

Where, *S*_abs_–absorbance of sample, *B*_abs_–absorbance of extract blank, and *C*_abs_–absorbance of the control (DPPH).

### Determination of Reducing Power Potential

Reducing power potential (RPP) was done according to the method as described by Rai et al. (20). Briefly, 100 μl (2.5 mg/ml) of the extract was mixed with 900 μl phosphate buffer (0.2 M) followed by the addition of 900 μl freshly prepared potassium ferricyanide (1%). The reaction mixture was vortexed and incubated at 50°C for 20 min. After the incubation, 900 μl of 10% TCA was added and centrifuged at 6,000 g for 10 min. The collected supernatant (900 μl) was added to an equal volume of distilled water and FeCl_3_ (0.1%). Absorbance was taken at 700 nm using a UV-VIS spectrophotometer. The data were expressed in AAE/g of ascorbic acid.

### Effects of Cooking on TPC, TFC, and Antioxidant Property of *U. Dioica*

The leaves of the *U. dioica* were cooked with two methods preferred by local people such as (i) boiling and (ii) steaming. Leaves of *U. dioica* (250 g) were divided into 5 equal parts. One part was taken as control and two parts were taken for boiling and the rest two were taken for steaming. Briefly, two sets of 50 g of leaves were taken in 400 ml distilled water and were boiled, respectively, for 5 min (B5) and 10 min (B10). Similarly, two sets of 50 g leaves were subjected to steam cooking for 20 min (S20) and 40 min (S40). The cooked leaves were then oven-dried at 40°C and ground to fine powders, which were then extracted in MeOH for further evaluation. The MeOH extracts were analyzed for TPC, TFC, and antioxidant activities (DPPH scavenging and TAA) as described earlier.

### *In-vitro* Simulated Gastrointestinal Digestion of Cooked and Steamed *U. Dioica*

Simulation of GI digestion was performed *in-vitro* following standard protocol mentioned in Minekus et al. (21) with modifications wherever required. The boiled and steam-cooked *U. dioica* leaves were treated for sequential simulation of mouth, stomach, and small intestine digestion. The compositions of simulated salivary fluid (SSF), gastric fluid (SGF), and intestinal fluid (SIF) are given in Table 1. The oral digestion was done independently on 10 g of boiled and steam-cooked leaves which was mixed with 7 ml SSF followed by the addition of 1 ml α-amylase solution (1,500 U/ml in SSF), 50 μl CaCl_2_ (0.3 M), and 1.95 ml distilled water to attain a paste of food: SSF-1:1 (w/v). The reaction mixture was then incubated for 5 min at room temperature. To the oral bolus 15 ml SGF, 3.2 ml pepsin solution (25,000 U/ml in SGF), 10 μl CaCl_2_ (0.3 M), and 1.39 ml distilled water were added for the simulation of the gastric digestion. The pH of the reaction mixture was maintained at 3 and incubated in shaking conditions at 37°C for 120 min. Similarly, simulation of the intestinal phase was performed on the gastric chyme with the addition of 22 ml SIF, 40 ml pancreatin solution (1:4 w/v), 80 μl CaCl_2_ (0.3 M), 0.3 ml NaOH, and 2.32 ml distilled water and then incubated at 37°C for 120 min at pH 7. After the complete simulation of digestion, the digesta were centrifuged at 4,000 rpm for 45 min and the supernatants were collected. The leftover residues were extracted with MeOH (1:5 w/v) to prepare the extract. TPC, TFC, and antioxidant activities were determined in both cases.

**Table 1:** Composition of simulated digestive fluids used in this study.

**Simulated digestive fluids**	**Volume of chemicals used (in mL)**					
	**KCl (37.3 g/L)**	**KH_**2**_PO_**4**_ (68 g/L)**	**NaHCO_**3**_ (84 g/L)**	**NaCl (117 g/L)**	**MgCl_**2**_. 6H_**2**_O (30.5 g/L)**	**(NH_**4**_)_**2**_CO_**3**_ (48 g/L)**
SSF, pH 7	30.2	7.4	13.6	-	1.0	0.12
SGF, pH 3	13.8	1.8	25.0	23.6	0.8	1.0
SIF, pH 7	13.6	1.6	85.0	19.2	2.2	-

*SSF, simulated salivary fluid; SGF, simulated gastric fluid; SIF, simulated intestinal fluid*.

## Results and Discussion

**Figure 1:**
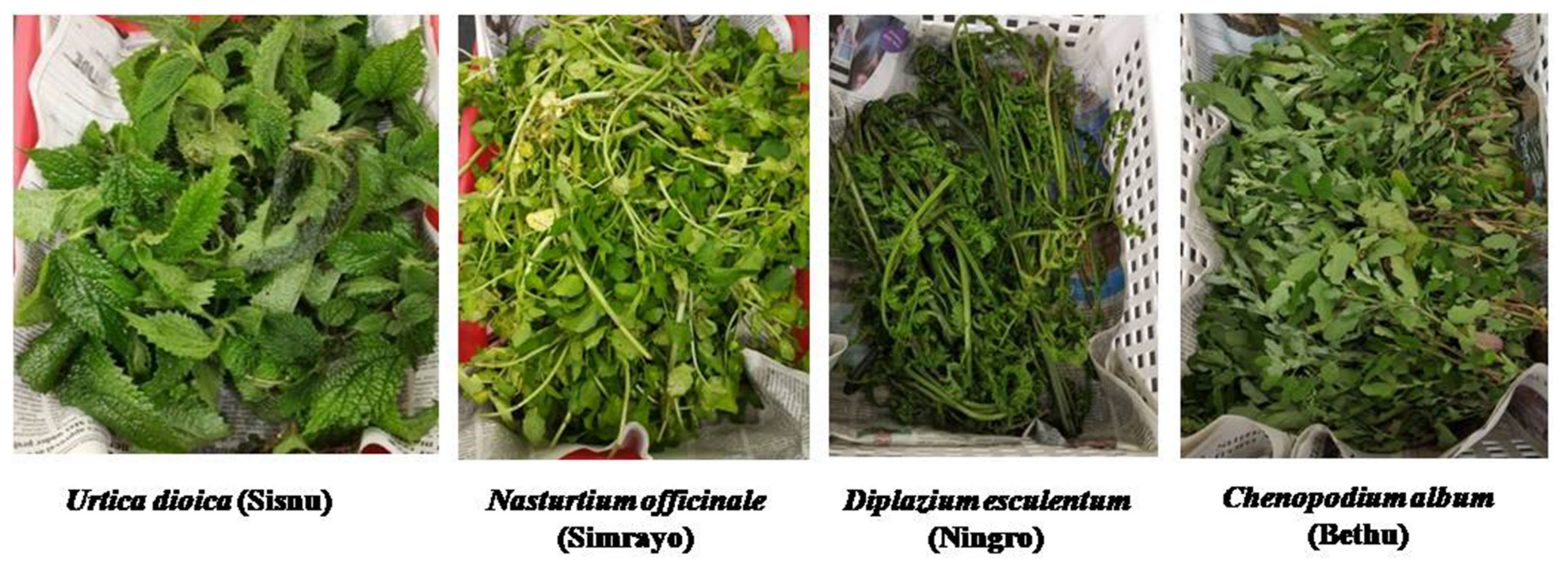
Selected green leafy vegetables popularly consumed in the Sikkim Himalayan region.

Leafy vegetables are essential sources of minerals, such as microelements, namely, K, Ca, Mg, P, and S, and microelements, namely, Fe, Cu, Mn, Zn, Na, Mo, and B, protein, dietary fiber, carbohydrates, and vitamins for human nutrition (22–26). Most importantly, they are rich in natural antioxidants such as phenolics, betalains, xanthophylls, violaxanthin, ascorbic acids, carotenoids, betacyanins, betaxanthins, chlorophyll A, chlorophyll B, and beta-carotene that have high radical quenching ability (27–31). Phenolics, the nonnutrient secondary metabolites found in fruits, seeds, and vegetables, have long been known for their biochemical and pharmacological importance. Phenolic compounds are considered to be vital in defense responses of the human body including anti-inflammatory, antiaging, antiproliferative, and antioxidative mechanisms (32). They include coumarins, phenolic acids, such as hydroxybenzoic acids and hydroxycinnamic acids, flavonoids, such as flavonols, flavones, flavanols, flavanones, isoflavones, anthocyanins, chalcones, and nonflavonoids, such as tannins, lignans, and stilbenes (33–37). Among others, flavonoids are the most prevalent and ubiquitous group of phenolic compounds that are widely distributed in fruits, plant-derived beverages, and vegetables. Mounting pieces of evidence in terms of epidemiological and clinical findings support their health-promoting and disease-preventing significance (38). Herein, selected GLVs consumed popularly in the SHR such as *U. dioica, N. officinale, D. esculentum*, and *C. album* (Figure 1) were studied for the determination of their TPC, TFC, and *in-vitro* antioxidant potential. The extract yield of MeOH, EtOAC, and hexane extracts of GLVs ranged from 3.97 to 8.05%, 1.77 to 4.50%, and 2.10 to 3.90% (dry weight basis), respectively (Table 2). Extract yield was found to be comparatively higher in MeOH extracts than that of EtOAC and hexane extracts. The TPC of GLV MeOH extracts ranged from 22.73 to 52.06 μg GAE/mg, 10.16 to 32.76 μg GAE/mg, and 9.14 to 25.40 μg GAE/mg of extract, respectively. Similarly, TFC of GLV organic extracts ranged from 2.93 to 11.18 μg QE/mg of extract in the MeOH, 3.42 to 14.86 μg QE/mg of extract in EtOAC, and 1.87 to 10.84 μg QE/mg of hexane extracts (Table 3). The *N. officinale* MeOH extract among others had the highest TPC (52.06 ± 3.82 μg GAE/mg of extract) followed by that of the *U. dioica*–MeOH extract (45.84 μg GAE/mg of extract). The *C. album* hexane extract was found to have the lowest estimate of TPC (9.14 ± 0.38 μg GAE/mg of extract). The *U. dioica* EtOAC extract was found to have the highest TFC (14.86 μg QE/mg of extract) followed by that of the MeOH (11.18 μg QE/mg of extract) and hexane extracts (10.84 μg QE/mg of extract). TFC was estimated to be the lowest in the hexane extract of *D. esculentum* (1.87 μg QE/mg of extract). The overall results showed variation in the TPC and TFC among different organic extracts of the same GLV.

**Table 2:** Scientific nomenclature and extract yield of selected leafy greens of Sikkim Himalayan Region.

**Scientific names**	**Common names**	**Total yield (%)**		
		**MeOH**	**EtOAC**	**Hexane**
*Urtica dioica*	Sisnu	8.05 ± 0.15^a^	3.87 ± 0.23^a^	2.92 ± 0.18^a^
*Nasturtium officinale*	Simrayo	5.64 ± 0.46^b^	4.50 ± 0.31^b^	3.90 ± 0.33^b^
*Diplazium esculentum*	Ningro	3.97 ± 0.23^c^	4.10 ± 0.38^a,b^	2.87 ± 0.13^c^
*Chenopodium album*	Bethu	4.80 ± 0.41^d^	1.77 ± 0.14^c^	2.10 ± 0.08^d^

*MeOH, methanolic Extract; EtOAC, ethyl acetate Extract; Hexane, hexane extract. Data expressed as mean ± standard deviation; different alphabets (a, b, c, and d) within the same column indicates significantly different at p < 0.05*.

**Table 3:** TPC and TFC in selected wild green leafy vegetables of Sikkim Himalayan region.

**Green leafy vegetables**	**TPC (μg GAE/mg of extract)**			**TFC (μg QE/mg of extract)**		
	**MeOH**	**EtOAC**	**Hexane**	**MeOH**	**EtOAC**	**Hexane**
*Urtica dioica*	45.84 ± 4.04^a^	22.86 ± 2.50^a^	15.87 ± 0.96^a^	11.18 ± 0.42^a^	14.86 ± 0.66^a^	10.84 ± 0.44^a^
*Nasturtium officinale*	52.06 ± 3.82^a^	32.76 ± 0.66^b^	25.40 ± 3.33^b^	3.32 ± 0.47^b,c^	5.02 ± 0.10^b^	7.20 ± 0.38^b^
*Diplazium esculentum*	31.11 ± 2.86^b^	16.76 ± 2.75^c^	11.05 ± 1.90^c^	2.93 ± 0.08^b^	3.42 ± 0.15^c^	1.87 ± 0.22^c^
*Chenopodium album*	22.73 ± 1.88^c^	10.16 ± 0.88^d^	9.14 ± 0.38^c^	3.61 ± 0.37^c^	4.15 ± 0.63^c^	2.50 ± 0.37^d^

*TPC, total phenolic contents; TFC, total flavonoid contents; MeOH, methanolic extract; EtOAC, ethyl acetate extract; Hexane, hexane extract. Data expressed as mean ± standard deviation; GAE, gallic acid equivalent; QE, quercetin equivalent; different alphabets (a, b, c, and d) within the same column indicate significant difference at p < 0.05*.

Biosynthesis and accumulation of phenolic compounds are appeared to be unconventional in each plant and plant organ, and variation in their contents relies on the growth stage and the genotypic composition of the plant species (39). The extractability and recovery of a particular organic component are reported to depend on the polarity of the extraction medium and the ratio of solute to solvent. Likewise, the extractability of phenolic compounds is reliant on the type and polarity index (PI) of the solvents used, and the solubility of the phenolic compounds in the solvents. In addition, their solubility is contingent on several chemical features including the position of –OH groups, their molecular size, and length of the hydrocarbon chains (40). Polar solvents are often considered suitable to extract most of the phenolic compounds from plant tissues (41, 42). MeOH has been found to be more efficient in extracting high content of low molecular weight phenolics from different plant parts (43). In contrast, flavonoids have an affinity toward both the polar and nonpolar extracting medium because of their diverse chemical structure (O-glycosides and aglycones). Polar solvents have been reported to extract the flavonoid glycosides while nonpolar solvents mostly extract their aglycones (44, 45). Resembling the above-mentioned facts, extracts of GLVs used in the study which were prepared using MeOH and EtOAC (polar extracting medium) appeared to have higher TPC and TFC, respectively. Quite the opposite, TFC in the *N. officinale* hexane extract was found to be higher as compared to other extracts and this can be certainly due to the presence of aglycones in the extract.

**Figure 2:**
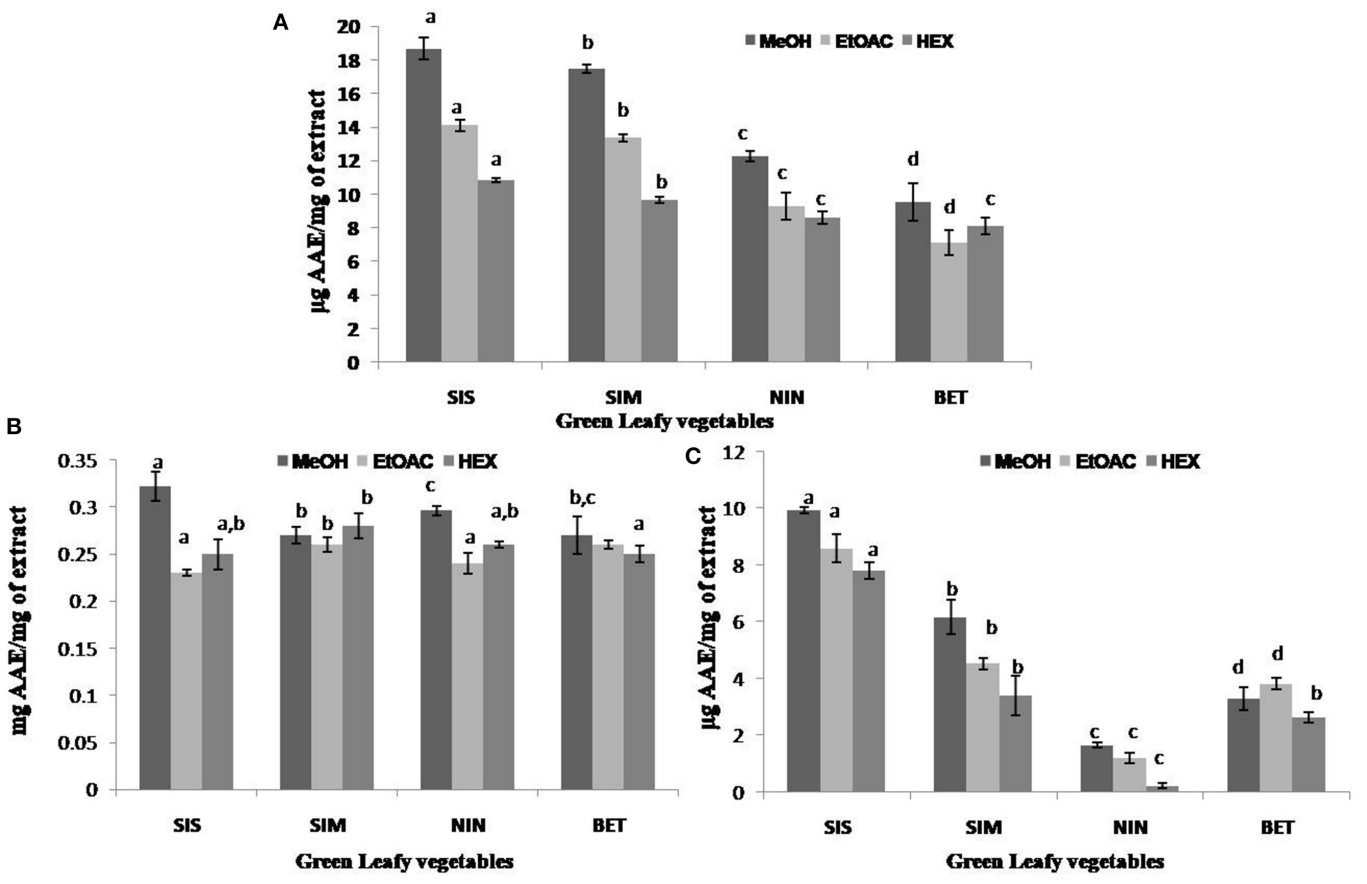
Antioxidant activities of different extracts of selected green leafy vegetables of Sikkim Himalayan region. **(A)** DPPH radical scavenging activity, **(B)** total antioxidant activity, and **(C)** reducing power potential. SIS, Sisnu *(Urtica dioica)*; SIM, Simrayo *(Nasturtium officinale)*; NIN, Ningro *(Diplazium esculentum)*; BET, Bethu *(Chenopodium album)*; MeOH, methanolic extract; EtOAC, ethyl acetate extract; HEX, hexane extract. Values not sharing common alphabets within the same pattern are significantly different (*p* < 0.05).

The antioxidant activities of the GLV organic extracts were determined using DPPH radical scavenging, TAA, and RPP. Evaluation of antioxidant potential is carried out using different methods as each assay varies in principle and mechanism of action (17). DPPH is a free radical which is a widely applied and acceptable method to study the antioxidant potential of plant extracts (18, 46). The DPPH scavenging activity of the GLV organic extracts ranged from 9.55 to 18.68 μg AAE/mg in MeOH extract, 7.11 to 14.10 μg AAE/mg in EtOAC extract, and 8.09 to 10.85 μg AAE/mg in hexane extracts. Among the test extracts, *U. dioica* MeOH extracts exhibited the highest DPPH radical scavenging activity (18.67 μg AAE/mg of extract), while the *C. album* EtOAC extract exhibited the lowest activity (7.11 μg AAE/mg of extract) (Figure 2A). TAA of the test extracts ranged from 0.27 to 0.32 mg AAE/mg of extract in MeOH, 0.23 to 0.26 mg AAE/mg of extract in EtOAC, and 0.25 to 0.28 mg AAE/mg of extract in hexane extracts. Among different plants, TAA of the *U. dioica* MeOH extract was found to be higher (0.32 mg AAE/mg of extract), whereas that of the EtOAC extract exhibited the lowest TAA (0.23 mg AAE/mg of extract) (Figure 2B). TAA assay measures the ability of the extract to reduce molybdenum (VI) to molybdenum (V) in acidic condition (47). RPP is an antioxidant method commonly applied for evaluating the presence of reductants, which exhibit antioxidant activity by breaking down of free radical chain on the donation of hydrogen atoms (43). RPP of the test extracts ranged from 1.67 to 9.93 μg AAE/mg of extract in MeOH, 1.2 to 8.60 μg AAE/mg of extract in EtOAC, and 0.23 to 7.80 μg AAE/mg of hexane extracts. Furthermore, the highest RPP was exhibited by *U. dioica* MeOH extract (9.9 μg AAE/mg of extract) and *D. esculentum* hexane extract displayed the lowest RPP (0.23 μg AAE/mg of extract) (Figure 2C). The overall results showed that the organic extracts obtained from *U. dioica* demonstrated relatively significant TPC and TFC, and exhibited promising antioxidant activity. Moreover, the *U. dioica* MeOH extract among others was able to exhibit higher antioxidant activity. Higher antioxidant activity in the *U. dioica* MeOH extract could probably be correlated to higher TPC present in the same (48).

**Figure 3:**
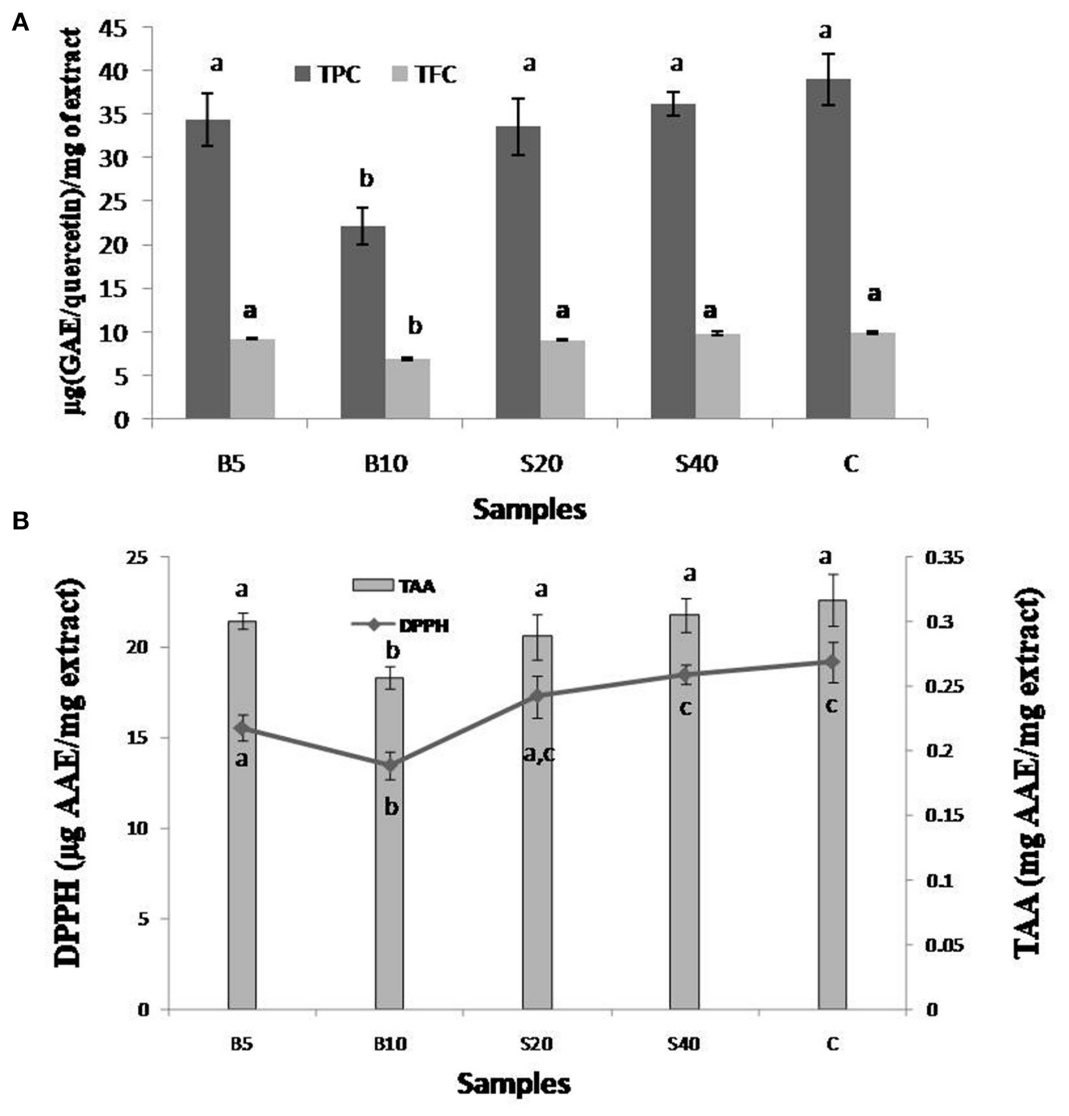
Effects of different cooking methods (steaming and boiling) on **(A)** phenolic (μg GAE/mg of extract) and flavonoid contents (μg QE/mg of extract) and **(B)** DPPH scavenging (μg AAE/mg of extract) and total antioxidant activity (mg AAE/mg of extract). TPC, total phenolic contents; TFC, total flavonoid contents; GAE, gallic acid equivalent; QE, quercetin equivalent; AAE, ascorbic acid equivalent. Values not sharing common alphabets within the same pattern are significantly different (*p* < 0.05).

The effects of cooking on the antioxidant potential of *U. dioica* leaf extracts were evaluated using steaming and boiling, two major methods used by the local people. Steaming of *U. dioica* leaves was carried out for 20 min (S20) and 40 min (S40), whereas boiling was carried out for 5 min (B5) and 10 min (B10) to enable proper cooking. The S40 extract of *U. dioica* demonstrated higher TPC (36.19 μg GAE/mg of extract) and TFC (9.84 μg QE/mg of extract) than S20 having 33.52 μg GAE/mg of extract and 9.04 μg Q/mg of extract, respectively. Conversely, B5 extract showed greater TPC (34.28 μg GAE/mg of extract) and TFC (9.23 μg QE/mg of extract) than B10 which is having TPC and TFC of 34.28 μg GAE/mg and 6.86 μg QE/mg of extracts, respectively. The results demonstrated an increase in TPC and TFC on steam cooking of *U. dioica* leaves (Figure 3A); however, loss of TPC and TFC was observed with leaf extracts prepared using boiled *U. dioica* leaves. The results resemble the findings made by Salamatullah et al. (49), Rocchetti et al. (50), and Gunathilake et al. (51). Similar observations were also witnessed while assessing the effects of cooking on the antioxidant activities (DPPH radical scavenging and TAA) given in Figure 3B. The S40 extract exhibited higher DPPH radical scavenging activity and TAA (18.47 μg AAE/mg of extract and 0.30 mg AAE/mg of extract) than that of the S20 extract (17.28 μg AAE/mg of extract and 0.28 mg AAE/mg of extract). In boiled cooking process, B5 extracts showed higher DPPH (15.54 μg AAE/mg of extract) and TAA (0.3 mg AAE/mg of extract) than B10 (DPPH 13.46 μg AAE/mg of extract and TAA 0.25 mg AAE/mg of extract). Higher boiling time reduced the antioxidant potential of *U. dioica*, as phenolics are released in water used for the boiling method. An increase in antioxidant activity on steam cooking may probably be due to the breakdown of complex structure and release of free from of phenolics on heat treatment (13). Several studies have shown steaming as a useful cooking approach over boiling to retain the antioxidant potential in a few vegetables (14, 49, 51). Therefore, it is essential to standardize the cooking procedure for individual leafy vegetable.

**Figure 4:**
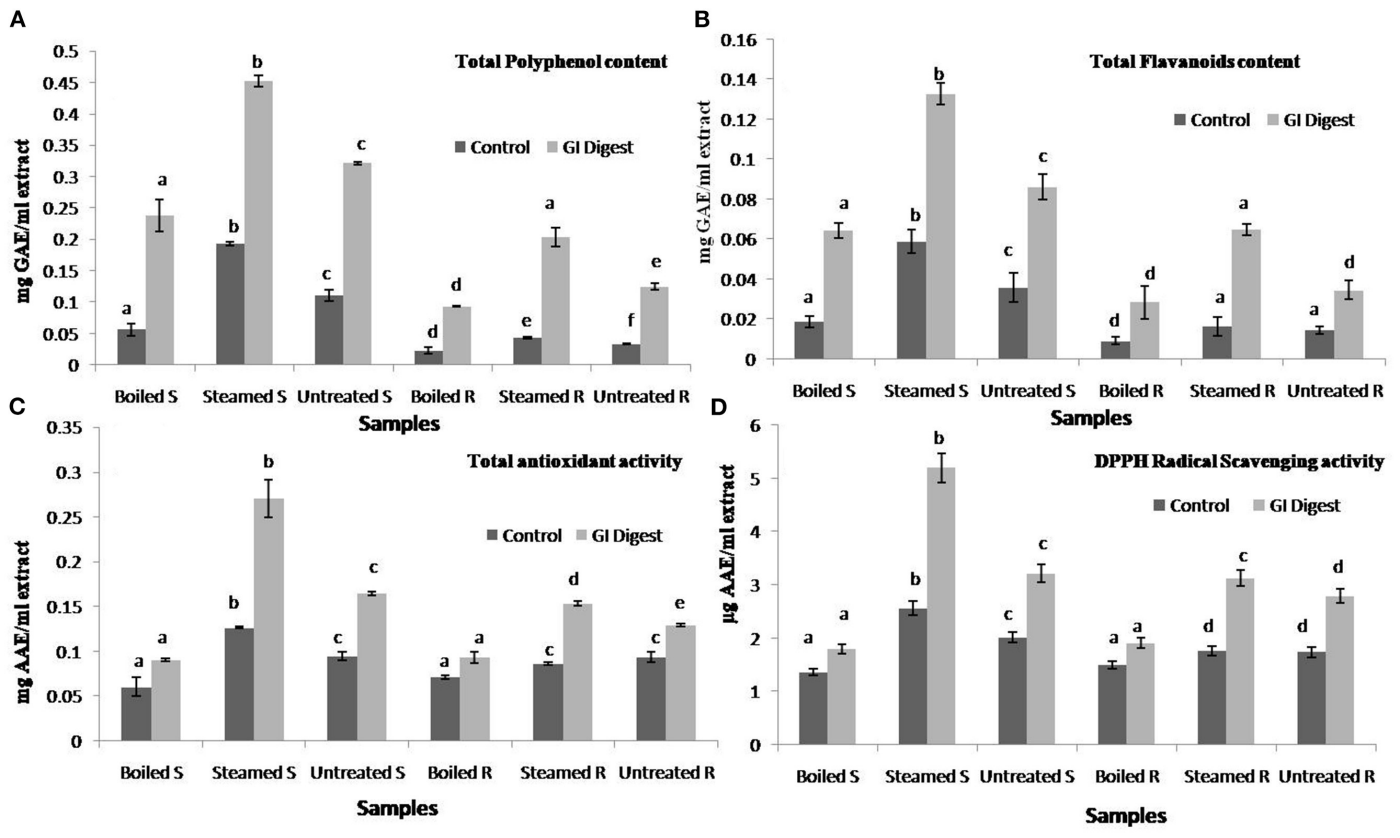
Effects of simulated gastrointestinal (GI) digestion on *Urtica dioica* leaves cooked by different methods (steaming and boiling) on **(A)** phenolics (mg GAE/ml of GI digested extract), **(B)** flavonoids content (mg QE/ml of GI digested extract), **(C)** total antioxidant activity (mg AAE/ml of GI digested extract), and **(D)** DPPH radical scavenging activity (μg AAE/ml of GI digested extract). Boiled S, boil cooked supernatant; Steamed S, steam-cooked supernatant; Untreated S, raw supernatant; Boiled R, boiled cooked residue extract; Steamed R, steam-cooked residue extract; Untreated R, raw residue extract. Values not sharing common alphabets within the same pattern are significantly different (*p* < 0.05).

The antioxidant effect of phytochemicals in GLVs will depend not only on their concentration but also on their resistance to the GI environment (13). The sequence of events during cooking followed by GI digestion may lead to an increase or decrease in antioxidant activity (1, 52). Simulated GI digestion of the steam-cooked and boiled leaves was performed using standard referred protocols, and TPC, TFC, and the antioxidant potential (DPPH scavenging and TAA) of the digesta (supernatants and residues) in each case were determined. TPC and TFC were estimated to be higher in steam-cooked and GI digested extracts (0.45 mg GAE/ml extract and 0.13 mg QE/ml extract, respectively) as compared to those estimated in boiled and GI digested (0.24 mg GAE/ml extract and 0.06 mg QE/ml extract, respectively) (Figures 4A,B). Similar findings were also observed in the case of TAA and DPPH scavenging activities where the extracts obtained from the steam-cooked and digested *U. dioica* leaves displayed higher activity (0.27 mg AAE/ml and 5.19 μg AAE/ml of extracts, respectively) as compared to the boiled and GI digested leaves (Figures 4C,D). The overall findings of the study suggest that the leafy vegetables consumed in the SHR could be a great source of natural antioxidant metabolites, namely, phenolics and flavonoids. In addition, steam-cooked *U. dioica* leaves could be preferred for consumption to ensure greater bioavailability of its phenolics and higher antioxidant effects. Furthermore, the findings may encourage the inhabitants of the SHR and other parts of the eastern Himalaya to use particular cooking approaches while cooking *U. dioica* leaves to help retain the phenolics and antioxidant properties.

## Conclusion

In this study, organic extracts of leafy vegetables commonly used by the local people of the Sikkim Himalayan region were evaluated for their phenolic and flavonoid contents, and antioxidant activity. The MeOH extract of *U. dioica* leaves among others was estimated to have higher TPC, TFC, and displayed significant antioxidant activity. The effects of cooking methods and GI digestion on the TPC, TFC, and antioxidant activity demonstrated that steam-cooked and digested leaves retained greater TPC and TFC, and antioxidant activity. The findings of this study could serve as a source of information in promoting the consumption of leafy vegetables in the SHR region and in the use of a definite cooking process to retain their antioxidant properties. Moreover, further research on the chemical efficacies of steam-cooked *U. dioica* leaves can be evaluated *in vivo*.

## Data Availability Statement

The original contributions presented in the study are included in the article/supplementary material, further inquiries can be directed to the corresponding author/s.

## Author Contributions

SS: methodology, investigation, validation, formal analysis, visualization, and writing—original draft. SPad: methodology, investigation, visualization, and data curation. MK: methodology, investigation, and data curation. SPat: resources, writing—review and editing, and supervision. DS: conceptualization, resources, writing—review and editing, visualization, supervision, and project administration. All authors contributed to the article and approved the submitted version.

## Funding

The authors would like to acknowledge the Institute of Bioresources and Sustainable Development and Department of Biotechnology, Government of India for the financial support.

## Conflict of Interest

The authors declare that the research was conducted in the absence of any commercial or financial relationships that could be construed as a potential conflict of interest.

## Publisher's Note

All claims expressed in this article are solely those of the authors and do not necessarily represent those of their affiliated organizations, or those of the publisher, the editors and the reviewers. Any product that may be evaluated in this article, or claim that may be made by its manufacturer, is not guaranteed or endorsed by the publisher.

## References

[1] Hossain A Khatun MA Islam M Huque R. Enhancement of antioxidant Quality of Green leafy vegetables upon different cooking method. Prev Nutr Food Sci. (2017) 22:216–22. 10.3746/pnf.2017.22.3.216 29043220 PMC5642804

[2] Sarker U Oba S. Drought stress enhances nutritional and bioactive compounds, phenolic acids and antioxidant capacity of Amaranthus leafy vegetable. BMC Plant Biol. (2018) 18:258. 10.1186/s12870-018-1484-1 30367616 PMC6203965

[3] Sarker U Oba S. Catalase, superoxide dismutase and ascorbate-glutathione cycle enzymes confer drought tolerance of *A. tricolor*. Sci Rep. (2018) 8: 16496. 10.1038/s41598-018-34944-0 30405159 PMC6220278

[4] Sarker U Oba S. The response of salinity stress-induced *A. tricolor to growth, anatomy, physiology, non-enzymatic and enzymatic antioxidants*. Front Plant Sci. (2020) 11:559876. 10.3389/fpls.2020.559876 33178233 PMC7596248

[5] Gülçin I Oktay M Kireçci E. Küfrevioglu ÖI. Screening of antioxidant and antimicrobial activities of anise (*Pimpinella anisum L*) seed extracts. Food Chem. (2003) 83:371–82. 10.1016/S0308-8146(03)00098-0

[6] Scalbert A Manach C Morand C Rémésy C Jiménez L. Dietary polyphenols and the prevention of diseases. Crit Rev Food Sci Nutr. (2005) 45:287–306. 10.1080/1040869059096 16047496

[7] Randhawa MA Khan AA Javed MS Sajid MW. Handbook of Fertility, Nutrition, Diet, Lifestyle and Reproductive Health. Cambridge: Academic Press. (2015). p. 205–20. 10.1016/B978-0-12-800872-0.00018-4

[8] Tan BL Norhaizan ME Liew WPP Sulaiman RH. Antioxidant and oxidative stress: a mutual interplay in age-related diseases. Front Pharmacol. (2018) 9:1162. 10.3389/fphar.2018.01162 30405405 PMC6204759

[9] Arfin S Jha N Jha S Kesari K Ruokolainen J Roychoudhury S. Oxidative stress in cancer cell metabolism. Antioxidants. (2021) 10: 642. 10.3390/antiox10050642 33922139 PMC8143540

[10] Bacchetti T Turco I Urbano A Morresi C Ferretti G. Relationship of fruit and vegetable intake to dietary antioxidant capacity and markers of oxidative stress: a sex-related study. Nutrition. (2019) 61:164–72. 10.1016/j.nut.2018.10.034 30716560

[11] Zhang D Hamauzu Y. Phenolics, ascorbic acid, carotenoids and antioxidant activity of broccoli and their changes during conventional and microwave cooking. Food Chem. (2004) 88: 503-509. 10.1016/j.foodchem.2004.01.065

[12] Miglio C Chiavaro E Visconti A Fogliano V Pellegrini N. Effects of different cooking methods on nutritional and physicochemical characteristics of selected vegetables. J Agric Food Chem. (2008) 56: 139–47. 10.1021/jf072304b 18069785

[13] Gunathilake KDPP Ranaweera KKDS Rupasinghe HPV. Influence of boiling, steaming and frying of selected leafy vegetables on the in vitro anti-inflammation associated biological activities. Plants. (2018) 7:22. 10.3390/plants7010022 29547518 PMC5874611

[14] Preti R Rapa M Vinci G. Effect of steaming and boiling on the antioxidant properties and biogenic amines content in green bean (*Phaseolus vulgaris*) varieties of different colors. J Food Qual. (2017) 2017:5329070. 10.1155/2017/5329070

[15] Dasgupta S. Effect of cooking on total phenol, total flavonoid, DPPH free radical scavenging assay and total antioxidant capacity of some green leafy vegetables commonly consumed in India. Indian J Appl Res. (2021) 11: 1–3. 10.30106/ijar

[16] Pradhan S Tamang JP. Ethnobiology of wild leafy vegetables of Sikkim. Indian J Tradit Knowl. (2015) 14:290–7.

[17] Rai AK Prakash M Anu Appaiah KA. Production of Garcinia wine: changes in biochemical parameters, organic acids and free sugars during fermentation of Garcinia must. Int J Food Sci Tech. (2010) 45:1330–6. 10.1111/j.1365-2621.2010.02181.x

[18] Aryal S Baniya MK Danekhu K Kunwar P Gurung R Koirala N. Total phenolics content, flavonoid content and antioxidant potential of wild vegetables from Western Nepal. Plants. (2019) 8:1–12. 10.3390/plants8040096 30978964 PMC6524357

[19] Rai AK Anu Appaiah KA. Application of native yeast from Garcinia (*Garcinia xanthochumus*) for the preparation of fermented beverage: changes in biochemical and antioxidant properties. Food Biosci. (2014) 5:101–7. 10.1016/j.fbio.2013.11.008

[20] Rai AK Jini R Swapna HC Baskaran V Sachindra NM Bhaskar N. Application of native lactic acid bacteria for fermentative recovery of lipids and proteins from fish processing waste: Bioactivities of fermentation products. J Aquat Food Prod Technol. (2011) 20:32–44. 10.1080/10498850.2010.528174

[21] Minekus M Alminger M Alvito P Ballance S Bohn T Bourlieu C. A standardised static in vitro digestion method suitable for food-An international consensus. Food Funct. (2014) 5: 1113–24. 10.1039/C3FO60702J 24803111

[22] Chakrabarty T Sarker U Hasan M Rahman MM. Variability in mineral compositions, yield and yield contributing traits of stem amaranth. (Amaranthus lividus) Genetika. (2018) 50:995–1010. 10.2298/GENSR1803995C

[23] Sarker U Islam MT Rabbani MG Oba S. Genotypic diversity in vegetable amaranth for antioxidant, nutrient and agronomic traits. Indian J Genet Pl Br. (2017) 77:173–6. 10.5958/0975-6906.2017.00025.6

[24] Sarker U Oba S. Protein, dietary fiber, minerals, antioxidant pigments and phytochemicals, and antioxidant activity in selected red morph *Amaranthus* leafy vegetable. PLoS ONE. (2019) 14: 222517. 10.1371/journal.pone.0222517 31830064 PMC6907799

[25] Zihad SMNK Gupt Y Uddin SJ Islam MT Alam MR Aziz S Hossain M Shilpi JA Nahar L Sarker SD. Nutritional value, micronutrient and antioxidant capacity of some green leafy vegetables commonly used by southern coastal people of Bangladesh. Heliyon. (2019) 5: *e*02768. 10.1016/j.heliyon.2019.e02768 31768435 PMC6872803

[26] Ejoh SI Wireko-Manu FD Page D Renard CMGC. Traditional green leafy vegetables as underutilised sources of micronutrients in a rural farming community in south-west Nigeria I: estimation of vitamin C, carotenoids and mineral contents. South Afr J Clin Nutr. (2021) 34:40–5. 10.1080/16070658.2019.1652963

[27] Oboh G. Effect of blanching on the antioxidant properties of some tropical green leafy vegetables. LWT-Food Sci Technol. (2005) 38:513–7. 10.1016/j.lwt.2004.07.007

[28] Subhasree B Baskar R Laxmi Keerthana R Lijina Susan R Rajasekaran P. Evaluation of antioxidant potential in selected green leafy vegetables. Food Chem. (2009) 115:1213–20. 10.1016/j.foodchem.2009.01.029

[29] Sarker U Oba S. Augmentation of leaf color parameters, pigments, vitamins, phenolic acids, flavonoids and antioxidant activity in selected *A. tricolor* under salinity stress. Sci Rep. (2018) 8:12349. 10.1038/s41598-018-30897-6 30120319 PMC6098045

[30] Sarker U Oba S. Response of nutrients, minerals, antioxidant leaf pigments, vitamins, polyphenol, flavonoid and antioxidant activity in selected vegetable amaranth under four soil water content. Food Chem. (2018) 252:72–83. 10.1016/j.foodchem.2018.01.097 29478565

[31] Sarker U Oba S. Nutritional and bioactive constituents and scavenging capacity of radicals in *Amaranthus hypochondriacus*. Sci Rep. (2020) 10:19962. 10.1038/s41598-020-71714-3 33203902 PMC7673121

[32] Hano C Tungmunnithum D. Plant polyphenols, more than just simple natural antioxidants: oxidative stress, aging and age-related diseases. Medicines. (2020) 7:26. 10.3390/medicines7050026 32397520 PMC7281114

[33] Sarkar P Singh SP Pandey A Rai AK. Microbial production and transformation of polyphenols. In: Rai AK Singh SP Sacool CR Larroche C Pandey A editors. Technologies for Production of Nutraceuticals and Functional Food Products. Elsevier. (2022). p. 189–208. 10.1016/B978-0-12-823506-5.00005-9

[34] Sarker U Oba S. Antioxidant constituents of three selected red and green color *Amaranthus* leafy vegetable. Sci Rep. (2019) 9:18233. 10.1038/s41598-019-52033-8 31796754 PMC6890792

[35] de la Rosa LA Moreno-Escamilla JO Rodrigo-García J Alvarez-Parrilla E. Phenolic compounds. In: Yahia EM, editor. Postharvest Physiology and Biochemistry of Fruits and Vegetables. UK: Woodhead Publishing. (2019). p. 253–71. 10.1016/B978-0-12-813278-4.00012-9

[36] Sarker U Hossain MN Iqbal MA Oba S. Bioactive Components and Radical Scavenging Activity in Selected Advance Lines of Salt-Tolerant Vegetable Amaranth. Front Nutr. (2020) 7:587257. 10.3389/fnut.2020.587257 33330589 PMC7734134

[37] do Carmo MAV Granato D Azevedo L. Antioxidant/pro-oxidant and antiproliferative activities of phenolic-rich foods and extracts: a cell-based point of view. In: Granato D, editor. Advances in Food and Nutrition Research. Cambridge: Academic Press. (2021), p. 253–80. 10.1016/bs.afnr.2021.02.010 34507644

[38] Anandh Babu PV Liu D. Flavonoids and cardiovascular health. In: Watson RR, editor. Complementary and Alternative Therapies and the Aging Population. Cambridge: Academic Press. (2009), p. 371–92. 10.1016/B978-0-12-374228-5.00018-4

[39] Bhandari SR; Kwak JH Jo JS Lee JG. Changes in phytochemical content and antioxidant activity during inflorescence development in broccoli. Chil J Agric Res. (2019) 79:36–47. 10.4067/S0718-58392019000100036

[40] Iloki-Assanga SB Lewis-Luján LM Lara-Espinoza CL Gil-Salido AA Fernandez-Angulo D Rubio-Pino JL Haines DD. Solvent effects on phytochemical constituent profiles and antioxidant activities, using four different extraction formulations for analysis of *Bucida buceras* L. and Phoradendron californicum. BMC Res Notes. (2015) 8:396. 10.1186/s13104-015-1388-1 26323940 PMC4553924

[41] Johari M Khong HY. Total phenolic content and antioxidant and antibacterial activities of *Pereskia bleo*. Adv Pharmacol Sci. (2019) 2019:7428593. 10.1155/2019/7428593 30719037 PMC6334350

[42] Begić S Horozić E Alibašić H Bjelić E Seferović S Cilović Kozarević E. Antioxidant capacity and total phenolic and flavonoid contents of methanolic extracts of *Urtica dioica* L. by different extraction techniques. Int Res J Pure Appl Chem. (2020) 21:207–14. 10.9734/irjpac/2020/v21i2330319

[43] Do QD Angkawijaya AE Tran-Nguyen PL Huynh LH Soetaredjo FE Ismadji S Ju YH. Effect of extraction solvent on total phenol content, total flavonoid content, and antioxidant activity of *Limnophila aromatica*. J Food Drug Anal. (2014) 22:296–302. 10.1016/j.jfda.2013.11.001 28911418 PMC9354875

[44] De Luna SLR Ramírez-Garza RE Serna Saldívar SO. Environmentally friendly methods for flavonoid extraction from plant material: impact of their operating conditions on yield and antioxidant properties. Sci World J. (2020) 2020:6792069. 10.1155/2020/6792069 32908461 PMC7474796

[45] Awouafack MD Tane P Morita H. Isolation and Structure Characterization of Flavonoids. In: Justino GC, editor. Flavonoids. IntechOpen. (2017). 10.5772/67881

[46] Ganesan P Kumar CS Bhaskar N. Antioxidant properties of methanol extract and its solvent fractions obtained from selected Indian red seaweeds. Bioresour Technol. (2008) 99:2717–23. 10.1016/j.biortech.2007.07.005 17706415

[47] Adegbaju OD Otunola GA Afolayan AJ. Effects of growth stage and seasons on the phytochemical content and antioxidant activities of crude extracts of *Celosia argentea* L. Heliyon. (2020) 6:4086. 10.1016/j.heliyon.2020.e04086 32514483 PMC7267717

[48] Ishiwata K Yamaguchi T Takamura H Matoba T DPPH. Radical-scavenging activity and polyphenol content in dried fruits. Food Sci Technol Res. (2004) 10:152–6. 10.3136/fstr.10.152

[49] Salamatullah AM Özcan MM Alkaltham MS Uslu N Hayat K. Influence of boiling on total phenol, antioxidant activity, and phenolic compounds of celery (*Apium graveolens* L) root. J Food Process Preserv. (2021) 45:15171. 10.1111/jfpp.15171

[50] Rocchetti G Lucini L Chiodelli G Giuberti G Montesano D Masoero F. Impact of boiling on free and bound phenolic profile and antioxidant activity of commercial gluten-free pasta. Food Res Int. (2017) 100:69–77. 10.1016/j.foodres.2017.08.031 28888460

[51] Gunathilake KDPP Ranaweera KKDS Rupasinghe HPV. Changes of phenolics, carotenoids, and antioxidant capacity following simulated gastrointestinal digestion and dialysis of selected edible green leaves. Food Chem. (2018) 245:371–9. 10.1016/j.foodchem.2017.10.096 29287383

[52] Bhatt A Patel V. Antioxidant potential of banana: study using simulated gastrointestinal model and conventional extraction. Indian J Exp Biol. (2015) 53:457–61. 26245031

